# On the Identity of Variola, Varioloid, and Variola Vaccina; and on the Prophylactic Power of the Vaccine Disease

**Published:** 1846-03

**Authors:** Jno. S. Rohrer


*On the Identity of Variola, Varioloid, and Variola Vaccina; and on the prophylactic power of the Vaccine Disease.* By Jno. S. Rohrer, M.D.

Dr. R. M. Huston.

*Dear Sir,*—As the present epidemic of small-pox is attended with more than usual fatality, any information which may tend to the establishment of some facts at this particular juncture, may not prove uninteresting to the medical public. For this reason, I will submit to the readers of the Examiner some views which I have long entertained in regard to this important subject, and detail a few experiments in confirmation of the same, made as long ago as the years 1829 and 1830.

Very respectfully yours,
Jno. S. Rohrer.

*Chestnut Street*, Jan. 30th, 1846.

In the investigation of this subject, my attention will be chiefly directed to the establishment of the identity of the diseases mentioned in the title. But, in order to do this with effect, it will be necessary to inquire more particularly into the nature of varioloid. I will also make some observations, and offer one or two facts, in relation to the prophylactic power of the vaccine disease.

North America was not visited by the small-pox until the beginning of the seventeenth century. It made its first appearance in the colony of Virginia, and soon spread throughout the inhabited part of the North American continent.

From its history, the small-pox has always been regarded as a very formidable disease. Although subdued in part by the introduction of kine-pox, it is still considered one of the most destructive maladies in the catalogue of human diseases. In its varioloid form it is less malignant; but as it still possesses all the elements of small-pox in a modified form, it may, nevertheless, be considered as a serious if not dangerous complaint. During the last ten or fifteen years, this variety has at various times been more or less prevalent throughout the country. In the city and county of Lancaster, (Pennsylvania,) it was extremely common in the years 1829 and 1830. Indeed, more or less of it occurred in almost every family in that populous section. While engaged in this campaign, the majority of varioloid cases that came under my observation had previously undergone a course of the vaccine disease; but I never met with it in a subject that had been *recently and successfully vaccinated.* In a few cases only which came under my notice, the disease occurred the third and fourth year after vaccination. This circumstance may be attributed to the vaccine disease not having been fully developed in the system, or to peculiarity of temperament. But, that the varioloid is small-pox modified by previous vaccination, or some other modifying influence, is a matter now too generally conceded to require much proof. If I were to adduce all the evidence in favor of this position, I might bring forward the most respectable authority; but it is unnecessary to go into this detail. There is one fact on this head which seems to be conclusive, and that is, matter taken from a varioloid pustule, and introduced into the arm of an unprotected person, will produce genuine small-pox.

The late Dr. Carpenter, of Lancaster, was one of the first who contended for the identity of the two affections; and although opposed by some of the most respectable physicians at that time, he nevertheless confirmed his position by experiments, which proved fully satisfactory. Having adopted the precautionary measures used in inoculation, the doctor introduced the matter of varioloid into the arms of two children who were unprotected; the result was *confluent small-pox*, in one instance of very aggravated symptoms, from which the patient finally recovered.

Another experiment, however, made by a medical friend of Dr. C., did not terminate so happily. The matter of varioloid (from a very mild case) was incautiously introduced into the system without preparing the patient for its reception, or restricting him in diet, &c.; and *confluent small-pox* was produced, of which the patient died.

These are a few of the many cases which came under the doctor's notice, in which the matter taken from a mild case of varioloid, unaccompanied by secondary fever, produced small-pox in patients unprotected. According to Dr. Eberle, the varioloid is not a new disease, but has been observed and described for centuries past, under the different names of *chicken-pox*, *horse-pox* and *swine-pox*.

*Variola Vaccina.*—In considering the subject of kine-pox, I shall endeavor, first, to give my views in regard to the origin and nature of the disease; secondly, the nature and extent of its prophylactic power.

With respect to the origin of this disease, different opinions have been entertained. Dr. Jenner believed that it originated from a disease in the heel of the horse, which farriers have termed the *grease*. Others have maintained that the disease in the cow was the only and true source of kine-pox. Dr. Jenner has, however, proved conclusively that the fluid of the grease, when applied to the nipple or udder of the cow, was capable of exciting in that animal the kine-pox.

The experiments made by Dr. Loy, in order to ascertain the true origin of the cow-pox, leave no doubt that the matter of the cow-pox, and that of the grease, are of the same nature, and operate on the human system in the same manner. The result of his experiments would tend to confirm the accuracy of Dr. Jenner's investigations.

Having premised thus much, I will now venture to advance an opinion long entertained by myself, in regard to the origin of the vaccine disease, which is, that the *cow-pox* is the *variola* of the *human subject* in a modified form; and that it was originally excited in the cow in the form of *cow-pox*, and in the horse in the form of grease, by the direct application of *variolous contagion* to these animals.

The analogy between the variolous and vaccine pustules is so great, that Dr. Jenner himself was struck so forcibly by the resemblance, as to think it highly probable that the grease in the horse might be the source of small-pox. It is, therefore, a matter of no little surprise, that so profound an investigator as Dr. Jenner should not have conceived the idea of tracing it to the *variola of the human subject.* The doctor ingeniously demonstrated that the kine-pox was originally transferred from the cow to the human subject in the process of milking. Is it not equally probable that the *small-pox* was originally propagated to the cow and horse in the same manner? For example; the dairy maid being affected with small-pox pustules, incautiously bears her part in milking, and thus communicates the disease to the cow; in the same way the ostler infects the horse in the act of dressing the heel. The disease being thus transmitted to these animals, not only undergoes a great modification, but has also acquired a new character, and is no longer communicable from one individual to another through the medium of the atmosphere.

In support of the above views, while residing in the country, in 1829, I inoculated several cows with small-pox matter, but, owing to the imperfect insertion of the matter, on account of the refractory disposition of the animals, or, what is more probable, to the cows having previously had the kine-pox, I did not fully succeed. In one instance only the animal was affected with inflammation and slight tumefaction. But, in order to set my position in a more favourable light, I will adduce in support of this doctrine an experiment performed by the late Dr. Carpenter, of Lancaster. Several years ago the doctor introduced the variolous matter, by inoculation, into the system of the cow, and the result, as he informed me, was a *perfect vaccine pustule.*

As it is an object of the greatest importance to ascertain the true source of the vaccine disease, I beg leave to suggest the propriety of *inoculating the cow with small-pox virus*, and permitting the matter to pass through different cows, until it shall be sufficiently modified to warrant the safety of its introduction into the human subject. Should this process be successful, as we have reason to hope, we can, at will, produce a modified disease which may prove the ultimate extinction of one of the most disastrous and deadly maladies that ever infested the human family!

If we reason from analogy, we are strongly supported in the opinion, that the diseases which we have been considering are essentially the same—apart from our present subject, the annals of medicine do not present an instance in which the influence of one disease, will secure the system from future attacks of another and a different disease. This anomaly, it would appear, is presented in vaccination alone. Should we not then be cautious in adopting a doctrine so much at variance with an established law? We should at least suspect it, until it had been brought to the test of repeated and close experiment.

In regard to the nature and extent of the prophylactic power of kine pox, I will now offer a few remarks, and adduce such facts as have been noticed by me during the late epidemic, as well as detail a number of experiments made in the years 1829 and 1830.

Dr. Jenner, as well as the most eminent physicians since his time, maintained the doctrine, that vaccination when successfully performed, is a permanent security against the infection of small pox. But, notwithstanding the high authority of Dr. Jenner, I do not believe that vaccination, however successfully performed, is a perfect security against future attacks of small pox, in all cases, unless the *operation of vaccination is from time to time resorted to.* That it wears out of the system is a fact which I believe can be fully and satisfactorily proven. Let us for a moment reflect upon the numerous cases which of late years have presented themselves to our notice, that had been successfully vaccinated, ten or fifteen years previously, and what has uniformly been the result? Why, that nearly the one-third of those who were exposed to the direct influence of variolous contagion, contracted the varioloid disease. In the years 1829 and 1830, while I resided in the city of Lancaster, the varioloid prevailed very extensively in that vicinity, so that very few families, as has been remarked, escaped the disease, although the precaution of vaccination throughout had been very strictly observed; but not in one instance did I observe a case in which it followed *recent and successful vaccination*. It would appear therefore from observation, that the susceptibility to variolous infection is regained in a series of years. During the epidemic above referred to, I was engaged in vaccinating a great number of children, and the following are some of the experiments I tried:

Having procured a fresh scab, which bore all the characteristic features of the genuine vaccine crust, I introduced the infection into the arms of eight infants, not exceeding the age of one year, that were unprotected. Every one of them received the genuine disease, which ran through its regular course. The same infants were revaccinated at the expiration of nine months after the first insertion; but no other effect ensued than a slight inflammation produced by the point of the lancet. Matter was now taken from the *same scab* which *failed in producing effect in the eight infants*, and introduced into the arms of a number of children between the age of eight and ten years, all of whom were vaccinated within a year of their birth by regular physicians, and in each of these it produced a *small pustule*, with slight efflorescence round the base, and some febrile excitement. At the same time a number of persons between the age of fifteen and twenty-five were revaccinated from the *infection used in the former instance*, and nearly all were affected more or less with the vaccine disease. I have it from respectable authority that these persons had all been vaccinated in their first and second years, and from the description I received, they all passed regularly through the disease; the vaccine pustules, however, in the above cases were not equally developed; some arrived at the highest point on the fifth day, and some on the sixth and seventh, whilst others not until the eighth and ninth days after the impression. It was seldom that the vaccine pustule after revaccination continued to increase up to the ninth day, except in persons of more advanced age. In individuals between the age of fifteen and eighteen, the disease was observed to be very regular until about the sixth day, after which it would decline and leave a circular scab, with a slight depression in the centre. But in those persons between the age of nineteen and twenty to twenty-five, the disease approached nearer to the regular form; and in a few instances it approached so near, that little difference if any could be observed; except that the disease was developed between the seventh and eighth day from the insertion. In a great majority of individuals who had been vaccinated fifteen or twenty years previously, the pustule of the revaccination came to the greatest perfection about the *sixth day*, and had evidently a spurious appearance. The above experiments were tried and noted down in the year 1829; but I can confidently assert that I have since revaccinated more than two hundred cases with similar results. In addition to the above, in the late epidemic, I revaccinated many old persons, and found, in the majority of cases, the disease approaching nearer to the genuine form than in younger ones.

From the foregoing facts and observations on this head, I think we may venture upon the following conclusion, viz: That all persons whose systems are in a state susceptible to taking the vaccine disease the second time, are also susceptible to the varioloid from the variolous contagion. I have never met or heard of a well authenticated case, in which the individual had taken the varioloid, after *recent and successful* vaccination; but hundreds and thousands have taken the disease ten, fifteen, and twenty years after the first impression of the kine pox. An infant will not take the regular vaccine disease more than *once in the same year.* We will venture to say that it is impossible to produce a second vaccine pustule in the same year. Thus we have a child secured against the varioloid influence in the first and second years after the impression of kine pox; but in eight or ten years more, its system is again susceptible to the influence of the variolous contagion. It does not seem that the vaccine disease does, in every instance, lose its prophylactic power; on the contrary, it is evident that in certain constitutions it affords complete protection against future attacks of the disease. But if the vaccine disease does not at all wear out of the system, to what should we attribute the frequent occurrence of the varioloid disease? It would be unreasonable to suppose that the affection affords complete protection forever after, and that the innumerable cases of varioloid which we daily witness, are owing to the carelessness or incompetency of physicians in performing the operation of vaccination.

The varioloid prevails most extensively in large cities, where vaccination is mostly performed by regular physicians. I attended on a family in the city of Lancaster, in this State, consisting of parents and eight children, all of which had received the regular vaccine disease, the latter in the first and second year of their ages, except the youngest child, which was unprotected, and at the age of two years took the small pox, and died on the eighth day. At the same time the father and the two eldest sons took the varioloid. The father had been vaccinated thirty years previously, and the two sons sixteen. All the rest of the family escaped the disease, although two of the younger children, between the age of four and six years, slept in the same bed in which the diseased was confined.

From these different experiments and observations, I have arrived at the following conclusions:

1st. That the *varioloid disease is small pox modified by previous vaccination, or some other modifying influence.*

2d. That the *cow pox is the variola of the human subject in a modified form; and that it was originally excited in the cow, in the form of cow pox, and in the horse in the form of the grease.*

3d. That the *kine pox is capable, in a series of years, of losing its prophylactic power.*

